# Development and efficacy of ex vivo expanded autologous regulatory T cells for the treatment of amyotrophic lateral sclerosis

**DOI:** 10.3389/fimmu.2026.1854252

**Published:** 2026-07-02

**Authors:** Ya You, Huifang Zhu, Qian Zhang, Juntao Wang, Chao Dong, Zheng Yang, Jun Zhang, Yushang Zhang, Maoyan Zhang, Mingqi Lu, Changsheng Du

**Affiliations:** 1 Key Laboratory of Spine and Spinal Cord Injury Repair and Regeneration of Ministry of Education, Orthopaedic Department of Tongji Hospital, School of Life Sciences and Technology, Tongji University, Shanghai, China; 2 Shanghai Saierxin Biomedical Technology Co., Ltd., Shanghai, China

**Keywords:** ALS, autoimmune disease, immunology, neurodegenerative disease, Treg

## Abstract

Amyotrophic lateral sclerosis (ALS) is a fatal neurodegenerative disease with limited therapeutic options, in which neuroinflammation critically drives disease progression. Regulatory T cells (Tregs) exert potent immunosuppressive and neuroprotective effects, offering great potential for ALS treatment. However, clinical application of Treg therapy is hampered by low peripheral cell abundance and unstable expansion quality. Here, we established and optimized a GMP-grade protocol for sorting and expanding peripheral blood-derived Tregs, and validated cryopreserved apheresis products as feasikble starting materials. Although ALS patient-derived Tregs showed reduced expansion capacity compared with healthy donor counterparts, they maintained comparable purity, stable regulatory phenotypes, and robust immunosuppressive function. Transcriptomic analysis confirmed the lineage fidelity and low pro-inflammatory characteristics of expanded Tregs. Therapeutic efficacy was verified in SOD1G93A ALS and GvHD mouse models with delayed disease progression and relieved inflammation. This study provides standardized GMP manufacturing strategies and solid preclinical evidence to support the ongoing clinical trial (NCT06671236) and facilitate Treg immunotherapy translation for ALS.

## 1 Introduction

Amyotrophic lateral sclerosis (ALS) is a progressive, fatal neurodegenerative disease characterized by the gradual loss of both upper and lower motor neurons in the cerebral cortex, brainstem, and spinal cord. This leads to muscle weakness and paralysis in patients, with death typically occurring within 3 to 5 years after diagnosis (1). Its pathogenesis involves the complex interplay of multiple factors, including genetic mutations (e.g., SOD1, C9orf72) (2–4), protein homeostasis disorders (5), oxidative stress (6), mitochondrial dysfunction (7), and neuroinflammation (8).

Current FDA-approved therapies such as Riluzole (9), Edaravone (10), and Tofersen (11) only modestly prolong survival (2–3 months) or delay symptom progression and are far from meeting clinical needs. Chronic neuroinflammation is a core pathological driver, manifested by dysregulated immune responses within the central nervous system (CNS), including microglial activation, astrocyte hyperplasia, and peripheral T cell infiltration (12).

Studies have demonstrated that regulatory T cells (Tregs) play a key role in inhibiting neuroinflammation and promoting neuronal survival (13). Tregs are a subset of CD4^+^ T cells expressing the transcription factor FoxP3, accounting for about 5-10% of peripheral CD4^+^ T cells, which is essential for maintaining immune homeostasis and limiting autoimmune damage (14, 15). Treg cells exert neuroprotective effects by inhibiting pro-inflammatory cells, promoting the transformation of macrophages/microglia from pro-inflammatory to anti-inflammatory phenotypes, reducing neuroinflammatory responses (16–19).

The protective role of Treg in ALS has been confirmed by multiple studies, a genome-wide association study (GWAS) including 20,806 patients demonstrated that the frequency of Treg cells is negatively correlated with the risk of ALS, suggesting the potential for Treg cell therapy to reduce disease risk (20). Mechanistic studies and clinical evaluations involving 33 patients with ALS revealed that the Treg content in cerebrospinal fluid was inversely correlated with the rate of disease progression (R = -0.40, *P* = 0.002) (21); In ALS patients and animal models, Treg failure accelerated disease progression, while restoration of Treg function in mouse models reduced neuroinflammation and prolonged survival in mice (22); In SOD1G93A mice, Treg amplification significantly delayed the disease progression by maintaining motor neuron cell size, inhibiting spinal astrocyte/microglial activation, and upregulating neurotrophic factor expression (21). Clinical experiments further showed that Tregs can inhibit oxidative stress-related acute phase protein levels (23). Results from another study suggested that Treg combined with IL-2 may produce a trend of alleviating disease progression (24).

Peripheral blood-derived Tregs (PB-Tregs) represent the most clinically accessible source. Peripheral blood mononuclear cells naturally contain CD4^+^CD25^+^FoxP3^+^ Tregs with immunosuppressive functions, which constitute one of the primary natural sources of functional Tregs (25). Autologous reinfusion therapy using the patient’s own peripheral blood sorting amplification of Tregs completely avoided allogeneic immune rejection (graft-versus-host disease, GVHD) and host anti-graft response (HVG) with significant safety (26–28).

However, PB-Treg therapy still faces some technical bottlenecks, such as the low frequency of Tregs in peripheral blood, generally 5% to 10% (29, 30), the number of Treg cells obtained by direct sorting is much lower than the clinical treatment requirement.

## 2 Materials and methods

### 2.1 Treg sorting

#### 2.1.1 Streaming sorting (FACS)

PBMC cells were purified using CD25 magnetic beads. CD25^+^ cells use monoclonal antibodies against CD4, CD25, CD127 (all purchased from Biolegend Biosciences, USA). T cell subsets were sorted on the FACSAria III high-speed cell sorter (Miltenyi Biotechnology, Germany) using the following antibodies: CD4-FITC (clone OKT4), CD127-BV421 (clone A019D5), and CD25-PE (clone BC96). After sorting, CD4^+^CD25^+^CD127^low^ cells were analyzed, and the purity was routinely greater than 95%. In the discussion of the results, it is referred to as Protocol A.

#### 2.1.2 Magnetic bead sorting of human CD8^-^CD19^-^CD25^+^ Tregs

Sorting of regulatory T cells (Tregs): Peripheral blood mononuclear cells (PBMCs) were sorted by density gradient centrifugation (Ficoll/Hypaque) from leukapheresis products of healthy donors (after informed consent and according to a protocol approved by the local institution). PBMC cells were subjected to negative selection using antiCD8 and antiCD19 magnetic beads (Miltenyi Biotechnology, Germany), followed by enrichment of CD25^+^ cells with anti-CD25 magnetic beads using a column-based system (Miltenyi Biotechnology). This procedure is referred to as Protocol B in the results section.

#### 2.1.3 CD4^+^CD25^+^ regulatory T cell sorting kit

NonCD4^+^ PBMC cells were indirectly magnetically labeled using a biotin-conjugated antibody mixture as a primary antibody labeling reagent, followed by an anti-biotin monoclonal antibody conjugated to magnetic beads as the secondary labeling reagent. The labeled cells were then negatively removed by a MACS^®^ column placed in the magnetic field of the MACS^®^ sorting magnet. CD4^+^ CD25^+^ regulatory T cells were directly labeled by CD25 beads and obtained by MACS column positive sorting from pre-enriched CD4^+^ T cell components. After removing the column from the magnetic field, the CD4^+^CD25^+^ regulatory T cells retained by the magnetic beads were collected. In the discussion of the results, it is referred to as Protocol C.

#### 2.1.4 APH sorting (GMP method based on CliniMACS)

A GMP selection protocol based on CD8, CD19 bead depletion and CD25 bead enrichment Treg isolation method was performed from healthy donors and patients leukapheresis products as used for CliniMACS Treg isolation. Leukapheresis of healthy donor was collected from healthy donors after obtaining informed consent, under a protocol approved by the Ethics Committee of Shanghai Zhaxin Integrated Traditional Chinese and Western Medicine Hospital (Approval No. 202423). Leukapheresis of patients was collected from patient after informed consent under a protocol approved by the Ethics Committee of The First Affiliated Hospital of Zhengzhou University (ClinicalTrials.gov ID: NCT06671236). APH cells were washed with PBS/EDTA buffer (Miltenyi Biotechnology, Germany), supplemented with 0.5% HSA (Baxter AG, USA). AntiCD8 and antiCD19 coated CliniMACS beads were added, incubated for 30 minutes and washed. CliniMACS program 2.1 was run to deplete labeled cells. The labeling procedure was repeated with antiCD25 CliniMACS beads, and CliniMACS program 1.1 was run to enrich for CD25 positive cells.

### 2.2 Treg production (amplification)

Human CD8^-^CD19^-^CD25^+^ Treg cells were inoculated at a density of 1×10^6^/mL in either Optimizer Medium (Invitrogen, USA) or X-VIVO 15 Medium (LONZA, Switzerland) at a density of 1×10^6^/mL in research laboratories. The medium was supplemented with 5% EliteGro-Adv supplementation substitute (SR) (EliteCell, USA) or human platelet lysate (HPL) (Biotest, Germany) with or without 100 nM Rapamycin (Miltenyi Biotech, Germany). Cells were activated using Dynabeads CD3/CD28 Treg Xpander (Invitrogen, USA) or TransACT (Miltenyi Biotech, Germany) at defined bead:cell ratios. Expanded cells were used for further analysis at each restimulation until day 22 of expansion, the expanded cells were collected for subsequent analysis.

During GMP production, Treg cells were seeded at a density of 1×10^6^ cells/mL in GMP cell expansion bags using Optimizer GMP Medium supplemented with 5% SR EliteGro-Adv (containing 100 nM rapamycin and activated with Dynabeads CD3/CD28 Treg Xpander (bead-to-cell ratio = 4:1). Expanded Treg cells were harvested on day 22 or when they reached 2×10⁹ cells. A small number of cells were taken for safety and functional analysis.

### 2.3 Cryopreservation

After the final harvest, all batches must meet the set release criteria, including: (1) CD4^+^CD25^+^CD127^-^ cells ≥ 90% of the live cell population; (2) impurity cells ≤ 10% of the live cell population; (3) ≤ 100 residual beads per 3×10^6^ cells; (4) viability ≥ 70%; (5) sterile: no growth after 14 days (standard of the Chinese Pharmacopoeia); (6) endotoxin < 0.5 EU/mL; (7) mycoplasma: not detected; (8) the inhibition rate ≥ 60% (Treg-to-T responder ratio = 0.5:1). For cryopreservation, the cell pellet was collected by centrifugation and resuspended in cryoculture medium at a concentration that contains the required cell dose in 10 mL. The product was transferred to a freezer bag, cooled to -80 °C in a programmed cooling freezer, and then transferred to liquid nitrogen (vapor phase) for long-term storage.

### 2.4 Analytical method and associated instrumentation

#### 2.4.1 Flow cytometry

Flow cytometry studies were performed using standard procedures. Briefly, peripheral blood mononuclear cells (PBMCs) were incubated with surface against antigen antibodies (CD4, CD25, CD127) for 30 min and then washed. FoxP3 staining was performed using the Anti-Human FoxP3 Flow Detection Kit (BioLegend, USA), following the manufacturer’s instructions. Flow cytometry studies were performed on a FACS Calibur flow cytometer (Beckman) and analyzed using flow cytometry software.

#### 2.4.2 *In vitro* inhibition experiments and cytokine assay

Cryopreserved PBMC cells (Teff) were thawed and labeled with 10uM CFSE. 1×10 (5)/well of Teff cells were co-cultured with Tregs at different ratios (Tregs: PBMC = 1:1-0.25:1) in X-vivo 15 medium and stimulated by anti-CD3/CD28-coated beads (Invitrogen) in U-bottom 96-well plates. Cells were incubated at 37°C, 5% CO_2_, for 3 days. After harvest, proliferation of CFSE-labeled Teff cells was acquired by flow cytometry and analyzed with flow cytometer software. The suppressive capacity of Treg lines was evaluated as the percentage reduction in Teff proliferation in the presence of Tregs. The calculation was based on the proliferation of responder T cells alone compared with the proliferation of cultures also containing Treg cells. After the suppression assay, culture supernatants were collected for the measurement of IFN-γ levels (BD Biosciences).

#### 2.4.3 RNA isolation

Total RNA was extracted according to the manufacturer’s protocol using the Total RNA Extractor (Trizol) kit (cat. no. B511311, Biobio, China) and treated with RNase-free DNase I to remove genomic DNA contamination. RNA integrity was assessed using a 1.0% agarose gel. Subsequently, RNA mass and concentration were assessed using a NanoPhotometer spectrophotometer (IMPLEN, CA, USA) and a Qubit 2.0 fluorometer (Invitrogen). The high-quality RNA samples were then sent to Bioengineering (Shanghai) Co., Ltd. for library construction and sequencing.

#### 2.4.4 Library construction and sequencing

A total of 1 μg of RNA was used for each sample as the input material for RNA sample preparation. Sequencing libraries were generated using the VAHTS mRNA-seq V2 Library Construction Kit (for Illumina) according to the manufacturer’s instructions, with index sequences added to distinguish different samples. Briefly, mRNA was purified from total RNA using magnetic beads with oligo (dT). In VAHTSTM First Chain Synthesis Reaction Buffer (5×), divalent cations were used for fragmentation at high temperatures. First-stranded cDNA was synthesized using random hexameric primers and M-MuLV reverse transcriptase (RNase H-). This was followed by second-strand cDNA synthesis using DNA polymerase I and RNase H. The remaining protruding ends were converted to flat ends by exonuclease/polymerase activity. After the 3’ end adenylation of the DNA fragment, the linker was attached to construct the library. To optimize cDNA fragments with a length of 150–200 bp, the library fragments were purified using the AMPure XP System (Beckman Coulter, Beverly, USA). Then, 3 μL of USER enzyme (NEB, USA) was incubated with cDNA of the size selected and ligated for 15 min at 37 °C and another 5 min at 95 °C, followed by PCR. PCR was performed using Phusion high-fidelity DNA polymerase, universal PCR primers, and Index (X) primers. Finally, the PCR product (AMPure XP System) was purified and the library quality was evaluated on the Agilent Bioanalyzer 2100 System. The libraries were then quantified and mixed. Double-terminal sequencing of the library was performed on a NovaSeq sequencer (Illumina, San Diego, CA, USA).

#### 2.4.5 Align with reference genome

Clean reads were aligned to the reference genome using HISAT2 (version 2.0) with default parameters. Alignment results were statistically evaluated using RSeQC (version 2.6.1). Genome-wide distribution and structural features were assessed with Qualimap (version 2.2.1), and gene coverage ratios were analyzed using BEDTools (version 2.26.0).

#### 2.4.6 Expression analysis

Gene expression values for transcripts were calculated using StringTie (version 1.3.3b). Principal component analysis (PCA) and principal coordinate analysis (PCoA) were performed to reflect distances and differences between samples. The TPM value (transcripts per million) eliminates the effects of gene length and sequencing differences, facilitating direct comparison of gene expression between samples. DESeq2 (version 1.12.4) was used to identify differentially expressed genes (DEGs) between two samples. If the q-value ≤ 0.001 and Fold Change ≥2, genes were considered significantly differentially expressed. When the normalized expression of a gene was zero between two samples, its expression value is adjusted to 0.01 (because 0 cannot be plotted on a logarithmic plot). If the normalized expression of a gene is less than 1 in both libraries, the gene was not included in subsequent differential expression analysis. Gene expression differences were visualized by scatter plots, MA plots, and volcano plots.

### 2.5 Animal pharmacodynamics

#### 2.5.1 *In vivo* efficacy of human Tregs against hPBMC-induced GvHD in C-NKG mice

7.5 × 10^6^ cells/animal human PBMCs were transplanted into severely immunodeficient C-NKG mice (Cyagen Biosciences (Suzhou) Inc.) to establish a GvHD model. The GvHD animal experiment was conducted at Cyagen Biosciences (Suzhou) Inc. All animal experimental operations and animal welfare assurance were authorized, approved and audited by the Institutional Animal Care and Use Committee (IACUC) in Cyagen Biosciences (Suzhou) Inc., (approval number: TACU24-FY037). In this study, six mice per group were used. Treg cells prepared according to the above protocol were administered intravenously once weekly for three consecutive weeks at a dose of 1 × 10^7^ cells per mouse per infusion. To evaluate the improvement of Treg cells on survival, weight loss, and GvHD episodes in mice.

#### 2.5.2 *In vivo* efficacy of mouse Tregs on spontaneous mice with ALS

Highly purified mouse CD4^+^CD25^+^ Tregs were prepared from donor mouse splenocytes according to the manufacturer’s instructions (STEMCELL, Cat. #18783) prior to each infusion. (The ALS animal experiment was conducted at Gempharmatech Co., Ltd. All animal experimental operations and animal welfare assurance were authorized, approved and audited by the Institutional Animal Care and Use Committee (IACUC) in Gempharmatech Co., Ltd., with the Protocol Number of GPTAP20240918-5.) Male B6-hSOD1G93A transgenic mice (GemPharmatech Co., Ltd.) were randomly divided into 2 groups (control group and Treg, 15 mice/group), while B6-WT non-transgenic mice (no spontaneous ALS, 5 mice) were recruited as mice of the same sex and equivalent age as WT controls. The Treg group was given 1 × 10^6^ cells/mouse Treg cells intrathecally, while the control group mice were given the same volume of PBS. The test article was administered every two weeks for a total of three injections, on days 0, 14, and 28. No administration was performed in the WT group. Mouse clinical observations, Kono scores, weight recordings, and motor function (limb grid and time to fall from the rotating bar) were monitored during life experiments.

The results of the pharmaceutical study were expressed as mean ± standard error (Mean ± SEM), and the paired Student’s t-test was used to compare the differences between groups. *P* ≤ 0.05 was considered statistically significant.

Animal experimental model validation results were expressed as mean ± standard error (Mean ± SEM), and data were analyzed using Graphpad prism 9. For the continuous data, when the variance of the two groups of samples was the same, the student’s t-test was used to compare the differences. If the variance is inconsistent, the Mann-Whitney rank-sum test is used to compare the differences. For graded data, the Mann-Whitney rank-sum test was used to compare differences. Analysis of variance (ANOVA) test was used for multi-group comparisons. *P* < 0.05 was a significant difference. The graphing software is Graphpad prism 9.

## 3 Results

### 3.1 Development and optimization of CMC processes

#### 3.1.1 Establishment of sorting methods

**Figure 1:**
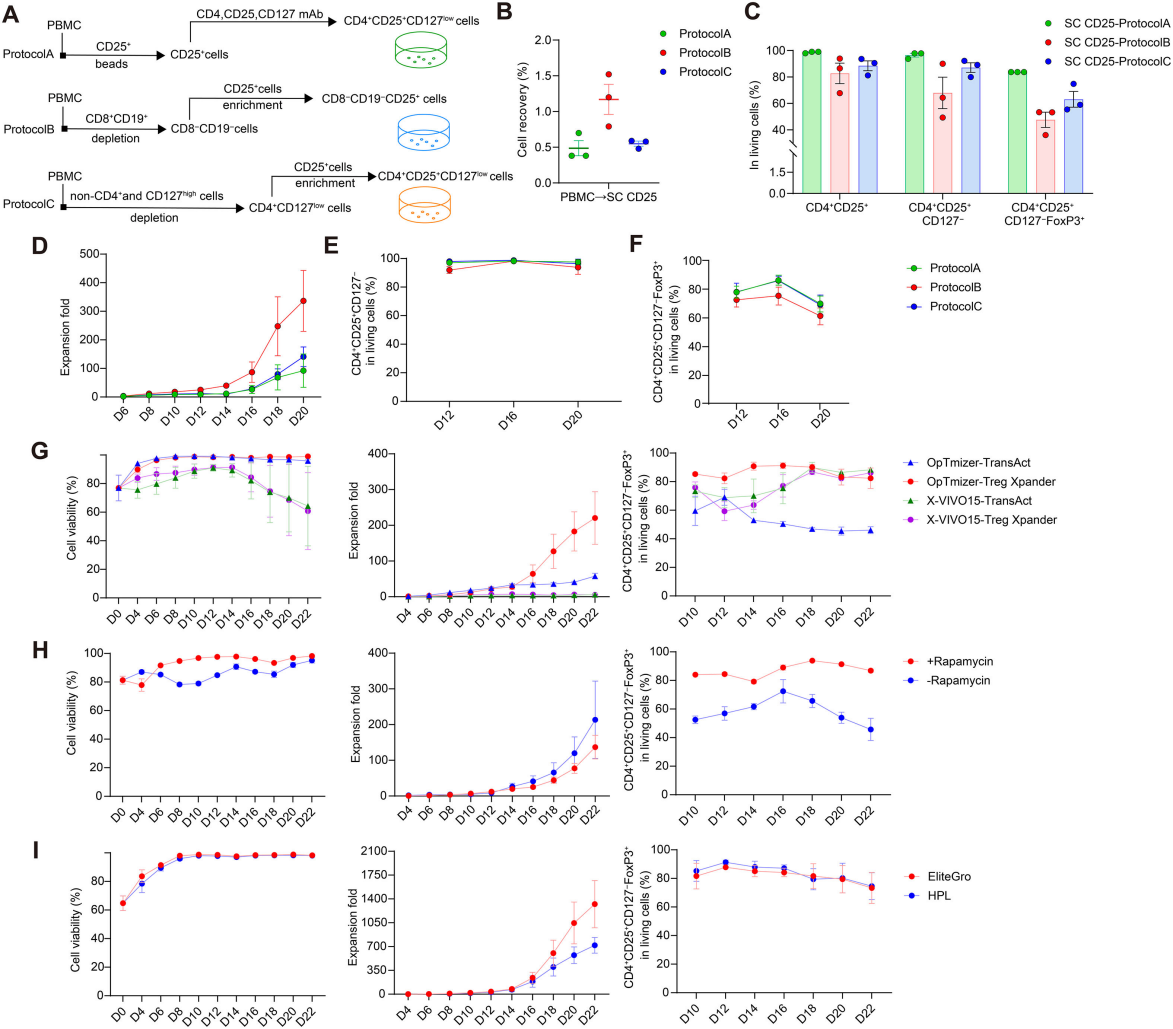
Differences in Treg cell products prepared by different cell separation methods (FACS-protocol A, microbeads-protocol B, kit-protocol C, during the sorting phase and *in vitro* expansion phase. n = 3, graphs show mean ± SEM. Sorting phase: **(A)** separation methods. **(B)** cell recovery rate. **(C)** sorting purity. **(D)** expansion fold. **(E)** The proportion of CD4^+^CD25^+^CD127^−^ Cells. **(F)** The proportion of CD4^+^CD25^+^CD127^−^FoxP3^+^ cells. Cell culture phase: **(G)** culture medium–activation reagent Combinations, n = 3, Graphs show Mean ± SEM. **(H)** Rapamycin intervention, n = 3, Graphs show Mean ± SEM. **(I)** Serum substitutes (EliteGro vs. HPL, Human platelet lysate), n = 5, Graphs show Mean ± SEM.

This experiment compares three cell sorting methods (n = 3) (**Figure 1A**). The results showed that Protocol B yielded the highest cell recovery (1.17% ± 0.21%), while Protocols A (0.49% ± 0.11%) and C (0.55% ± 0.04%) produced comparable yields (**Figure 1B**). Protocol A exhibited the highest cell viability (98.52% ± 0.26%). No significant differences in cell viability were observed among Protocol A, Protocol B (88.36% ± 2.86%), and Protocol C (92.00% ± 1.94%) after sorting (**Supplementary Figure 1**). The proportion of CD4^+^CD25^+^CD127^−^ cells of Protocol B was the lowest (67.94% ± 11.89%), and the proportion of CD4^+^CD25^+^CD127^−^ cells of Protocol A was the highest (96.5% ± 1.39%) (**Figure 1C**). As expected, higher purity was achieved following flow cytometric sorting, accompanied by a reduction in total cell yield. The amplification factor was the best in Protocol B, with an amplification factor of 442.89 ± 10.00 at D20 harvest. As the culture progressed, the proportion of CD4^+^CD25^+^CD127^−^ cells between the groups tended to be close (Protocol A: 97.55% ± 1.70%; Protocol B:93.83% ± 4.88%; Protocol C:96.26% ± 3.29%) (**Figures 1D–F**). It was proved that the culture conditions established in this experiment were only suitable for the proliferation of Treg cells. Due to the low yield of flow cytometry and the difficulty of meeting the strict GMP requirements, the Protocol C kit was non-GMP grade, so Protocol B can only be selected for formal GMP production.

#### 3.1.2 Establishment of *in vitro* amplification method

In this study, an *in vitro* culture protocol for Treg cells was systematically established, and the cell viability and expansion of medium type (OpTmizer, X-VIVO15), activation reagent (Treg Xpander, TransAct), rapamycin (Rapamycin) and serum substitute (EliteGro, HPL) were evaluated Effects of expansion and purity (CD4^+^CD25^+^CD127^−^FoxP3^+^). In the media reagent confirmation (n=3) study, the two groups using X-VIVO15 showed a significant decrease in viability after D12. Cell viability in the OpTmizer medium was more stable and consistently higher compared with the other culture conditions. The OpTmizer–Treg Xpander group demonstrated significantly greater expansion capacity than the other three groups, achieving a mean 220.50 ± 73.52-fold increase by Day 22 of culture. The proportion of CD4^+^CD25^+^CD127^−^FoxP3^+^Treg cells remained stable and was 82.38% ± 7.15% at D22 (**Figure 1G**). In the study of whether rapamycin was added (n=3), it was found that the cell viability of the +Rapamycin group was higher and more stable, although the amplification fold was lower than -Rapamycin group (D22, +Rapamycin group 136.88 ± 33.04 times vs. -Rapamycin group 213.46 ± 108.32 times), but the proportion of CD4^+^CD25^+^CD127^−^FoxP3^+^ was higher (at D22: 86.89% ± 1.19% vs. -Rapamycin group 45.73% ± 7.79%) (**Figure 1H**). The results of comparing serum substitutes (n=5) showed that the expansion efficiency of the EliteGro group was significantly higher than that of the HPL group (D22 amplification fold: 1323.17 ± 347.37 times vs. 718.26 ± 115.76 times), the proportion of CD4^+^CD25^+^CD127^−^FoxP3^+^ cells (D22: 73.31% ± 10.83% vs 74.57% ± 9.40%) had no significant difference (**Figure 1I**). In conclusion, OpTmizer-Treg Xpander combined with rapamycin and EliteGro can balance amplification efficiency and functional phenotypic retention in long-term culture, providing key parameters for GMP process optimization.

Stability studies are currently ongoing, with up to two batches of 15-month stability data available. The detection indicators include pH value, osmolarity, Treg purity (CD4^+^CD25^+^CD127^−^Foxp3^+^ cells), inhibitory function, cell viability, viable cell density, proportion of non-target cells (CD8^+,^ CD14^+^, CD19^+^, CD56^+^ cell sum), bacterial endotoxin examination, sterility examination and mycoplasma examination. Although fluctuations were observed in each detection item, no trends such as a significant decrease were noted, and all indicators complied with the quality standards. (**Supplementary Figure 2**).

### 3.2 Feasibility verification of APH after cryopreservation as raw material for production

**Figure 2:**
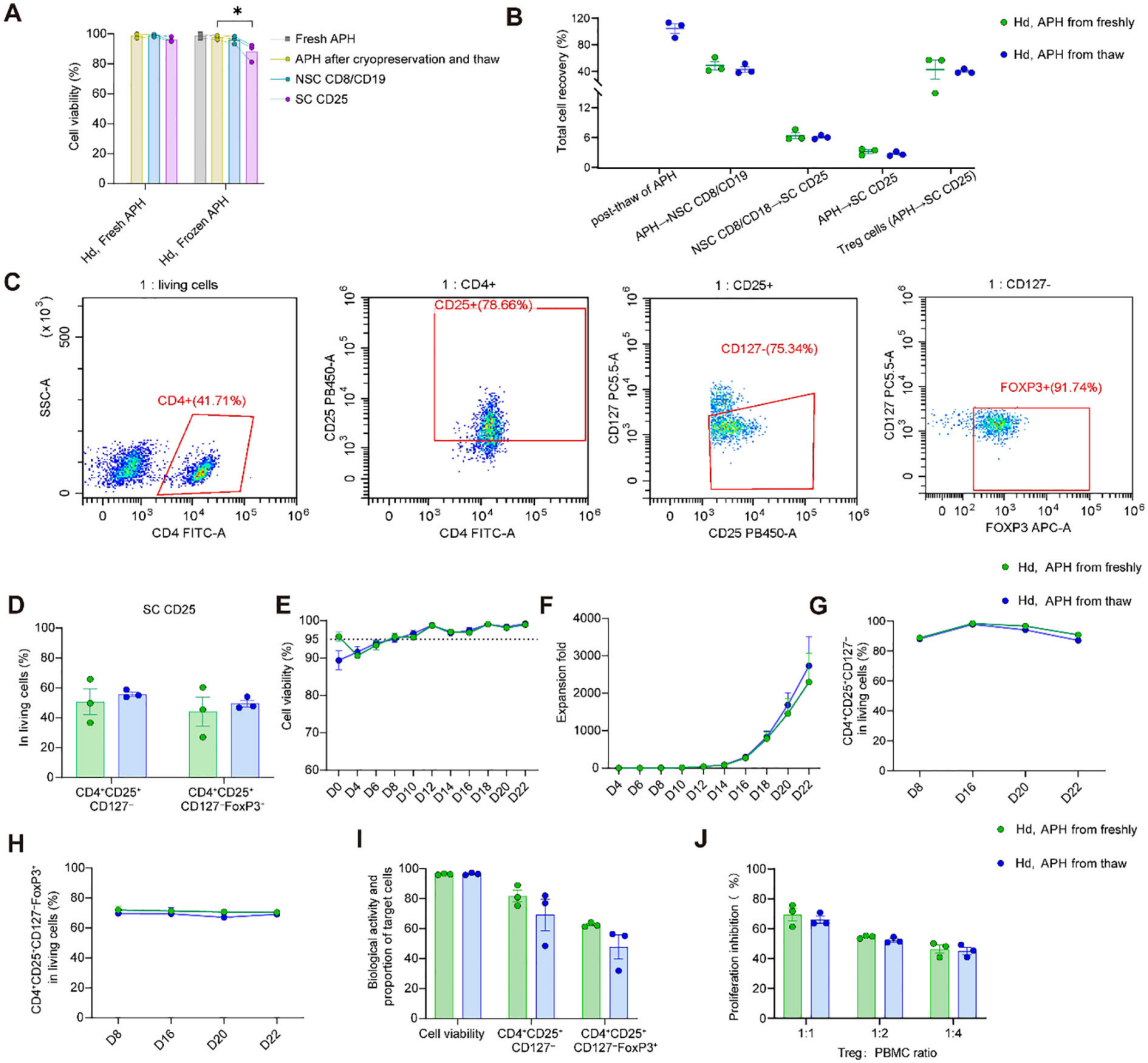
Differences in cellular biological properties and expansion efficiency of Treg cell products generated from fresh *vs.* thawed apheresis components **(APH)** cells of healthy donors as starting Cells. n = 3, Graphs show Mean ± SEM. Sorting Phase: **(A)** Cell viability. **(B)** Cell recovery. **(C)** D0 Flow cytometry example for SC CD25. **(D)** Treg purity. Culture Phase: **(E)** Cell viability. **(F)** Expansion fold. **(G)** CD4^+^CD25^+^CD127^−^Treg purity. **(H)** CD4^+^CD25^+^CD127^−^FoxP3^+^ Treg purity. Treg Cell Product: **(I)** Comparison of Treg cell viability and Treg purity. **(J)** Proliferation inhibition rate comparison at different Treg: peripheral blood mononuclear cell (PBMC) ratios. (*P<0.05).

When fresh APH products arrives at the production site, immediate processing may not always be feasible, and to preserve the possibility of secondary preparation, this study compared the differences in different states of APH in the sorting, culture and final product stages of healthy donors (APH from freshly, n=3; APH from thaw, n=3). The results showed that the cell viability of cryopreserved APH was slightly lower than that of the fresh APH group (88.13% ± 3.49% vs. 96.25% ± 0.98%) after the last step of sorting. There was no significant difference in cell recovery between cryopreserved APH and fresh group at each step. At the end of the final step of sorting, the proportion of CD4^+^CD25^+^CD127^−^ cells in the fresh group was 55.83% ± 1.50% vs. 57.04% ± 4.68% in the cryopreservation group, and the proportion of CD4^+^CD25^+^CD127^−^FoxP3^+^ cells in the fresh group was 46.74% ± 3.67% vs. 51.75% ± 4.71% in the cryopreservation group. There was also no significant difference (**Figures 2A–D**). To achieve the target Treg cell dose required for clinical reinfusion, the sorted cells were expanded *in vitro*. Both groups exhibited high viability, maintaining levels above 95% after D6 of culture, with nearly overlapping growth trends. In the early stage (D4-D14), both groups of cells were in the recovery period; D16 rapid amplification. At harvest, the proportion of CD4^+^CD25^+^CD127^−^ Treg cells in both groups was > 80%, and the proportion of CD4^+^CD25^+^CD127^−^Foxp3^+^ cells remained stable, and there was no difference between fresh and cryopreserved APH (**Figures 2E–H**). There was no significant difference between HD cryopreserved and fresh APH cells in terms of cell viability, expansion capacity, and purity, indicating that cryopreservation resuscitation of APH is a feasible starting material strategy.

The Treg cells cultured *in vitro* from the two starting sources of APH were finally cryopreserved, and the cell viability (mean > 96%) of the two groups was similar. The CD4^+^CD25^+^CD127^−^ Treg cells (69.25% ± 10.45%) of APH in the cryopreservation group were lower than those in the fresh APH group (81.84% ± 3.99%). The APH cryopreservation group of CD4^+^CD25^+^CD127^−^FoxP3^+^ Treg cells (47.89% ± 7.96%) was also lower than that of the fresh APH group (62.63% ± 0.85%). There was a decrease compared with Treg before cryopreservation, but there was no significant difference between groups (**Figure 2I**). The results showed that the inhibition rate of cryopreservation APH-derived Treg was 65.98% ± 2.4%, and the inhibition rate of fresh APH derived Treg was 69.54% ± 4.28% when the ratio of Treg and PBMC cells was1:1. As the proportion of Treg added decreases, the inhibition rate also decreases, showing a dose-response relationship (**Figure 2J**).

### 3.3 GMP manufacturing: ALS patients vs health donors

**Figure 3:**
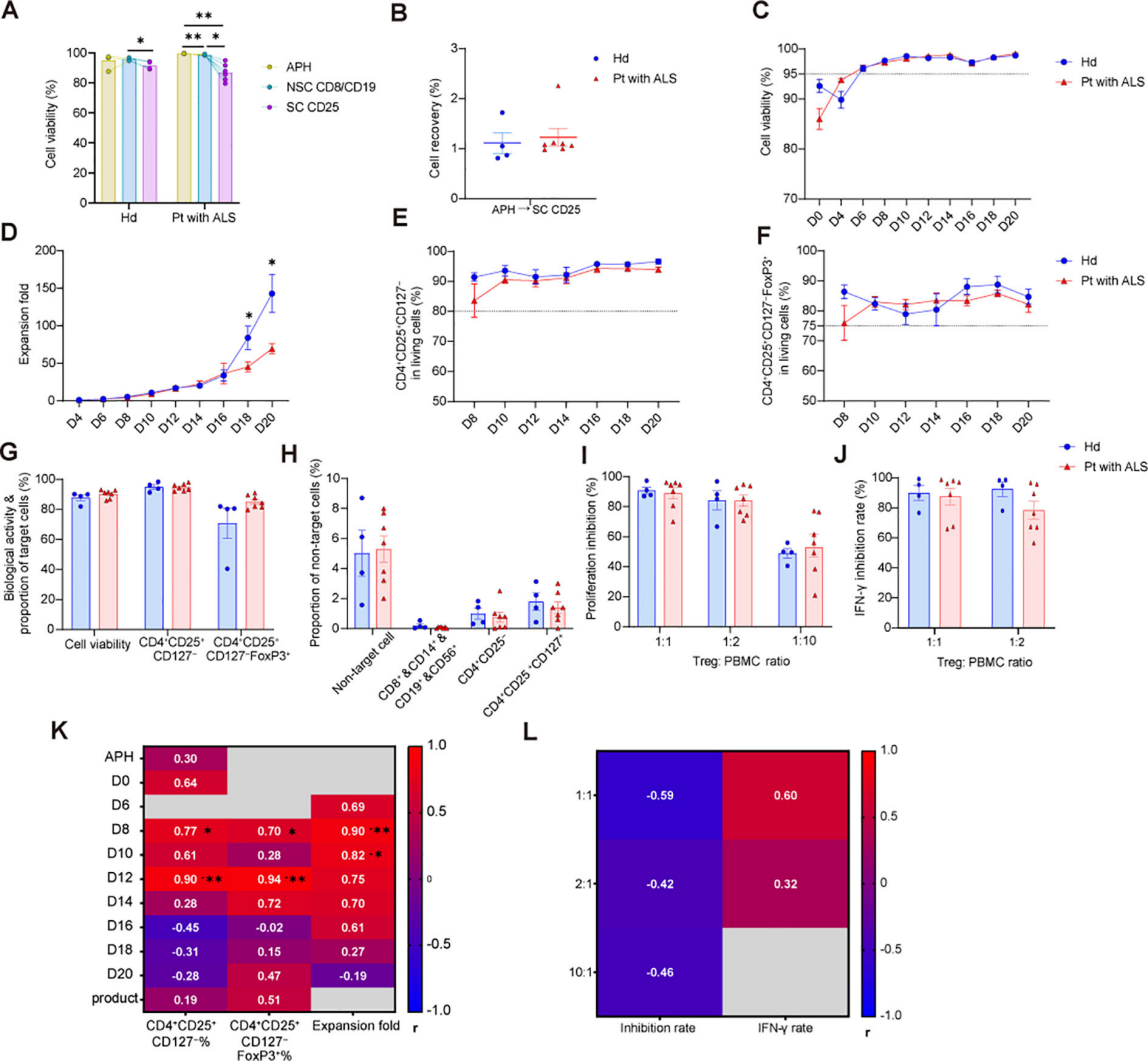
Differences in cell sorting and ex vivo culture during GMP manufacturing between healthy donors and patients with ALS. HD: n=4, ALS: n=7, graphs show mean ± SEM. Sorting phase: **(A)** Cell viability. **(B)** Cell recovery. Culture phase: **(C–F)** Cell viability, Expansion fold, CD4^+^CD25^+^CD127^-^Treg purity, CD4^+^CD25^+^CD127^-^FoxP3^+^Treg purity. Treg product after cryopreservation and thawing: **(G)** Cell viability, CD4^+^CD25^+^CD127^-^Treg purity, CD4^+^CD25^+^CD127^-^FoxP3^+^Treg purity. **(H)** Proportion of non-target cells. **(I, J)** Inhibitory effect of Treg on IFN-γ secretion by PBMCs at different Treg: PBMC ratios, graphs show Mean ± SEM. **(K, L)** Correlation analysis between key cell manufacturing parameters and ALSFRS-R scores in seven ALS patients, the data for IFN-γ comes from five ALS patients. (*P<0.05; **P<0.01).

To verify that patient-derived Treg cell products produced under GMP conditions exhibit the same quality and functionality as healthy donors during process development (**Figure 3**) and compared the quality differences in the manufacturing process and final product of Treg cell products in HD (n = 4) and ALS patients (n = 7). In the sorting stage, the total cell recovery of ALS patients was 1.13% ± 0.21%, HD:1.23% ± 0.17%, with no significant difference, and the mean cell viability of both groups were > 86% (**Figures 3A, B**). During the *in vitro* culture of Treg cells, the cell viability of the two groups after D6 recovered to more than 95%. At the time of cell harvesting, the cell viability of the HD group (D20 98.69% ± 0.13%) was comparable to that of the ALS group (D20 99.06% ± 0.13%). The amplification factor in the HD group (D20 142.99 ± 25.26) was significantly higher than that in the ALS group (D20 69.41 ± 6.70) (*p* < 0.05). (**Figures 3C, D**) The proportion of CD4^+^CD25^+^CD127^−^ Treg cells in the HD group was 96.61% ± 0.80%, and the proportion of ALS patients was 93.94% ± 0.83%, the difference between the two groups was not obvious. The proportion of CD4^+^CD25^+^CD127^−^FoxP3^+^ Treg cells in the HD group was 84.67% ± 2.45%, and the proportion of ALS patients was 82.20% ± 2.70%, there was also no significant difference between the two groups (**Figures 3E, F**). There were no significant differences in cell viability and CD4^+^CD25^+^CD127^−^cells between ALS patients and HD after cryopreservation resuscitation of Treg Product. The proportion of CD4^+^CD25^+^CD127^−^FoxP3^+^ Treg in the HD group (70.87% ± 10.14%) was lower than that in the ALS group (85.10% ± 1.65%), and there was no significant difference. The proportion of non-target cells remained low in both groups (**Figures 3G, H**).

*In vitro*, it can be observed that there was no significant difference in the inhibitory function of PBMCs amplification and the inhibition of IFN-γ secretion between ALS patients and HD-derived Tregs. And with the difference in the ratio of Treg: PBMCs, the dose-effect relationship is presented. The inhibition efficiency of ALS group (88.92% ± 3.75% at 1:1; 84.06% ± 3.80% at 1:2; 52.84% ± 7.64% at1:10) was basically the same as that of the HD group. The inhibition rate of IFN-γ secretion in the ALS group was 78.41% ± 5.95%, which was slightly lower than that in the HD group 87.51% ± 5.51%, but there was no significant difference (**Figures 3I, J**).

The relationship between ALS disease and Treg GMP manufacturing process-related parameters suggested that: ALSFRS-R score is a standardized tool to assess the functional status and rate of disease progression in ALS patients. In this study, 7 patients with ALS were subjected to correlation analysis with ALSFRS-R score (**Figures 3K, L**) based on the content of Treg cells in the initial APH, the expansion ability, the phenotype and inhibitory function of the final product Treg, and the core findings were as follows:

1. The ALSFRS-R score showed a weak positive correlation trend with the proportion of CD4^+^CD25^+^CD127^−^ of the starting APH cells of the patients, and a moderate positive correlation trend with the proportion of CD4^+^CD25^+^CD127^−^ of Treg cells after sorting.
2. Correlation between ALSFRS-R score and *in vitro* expansion status of Treg cells: During D8–D12, ALSFRS-R score showed a strong positive correlation with the proportion of CD4^+^CD25^+^CD127^−^ and CD4^+^CD25^+^CD127^−^FoxP3^+^, and there was a significant difference between D8 and D12 (*P* < 0.05). In the later stage of culture (after D16), this strong positive correlation was greatly weakened or the correlation direction is reversed (e.g., from positive to negative correlation trend). The correlation with the score also weakened as the amplification progresses. These results suggest that the higher the ALSFRS-R (the milder the disease) in ALS patients, the higher the proportion of Treg cells and the higher the cell expansion fold. The correlation results have important reference significance for cell expansion in clinical patients.
3. Correlation between ALSFRS-R score and the characteristics of Treg final products: ALSFRS-R score showed a positive correlation with the proportion of CD4^+^CD25^+^CD127^−^ and CD4^+^CD25^+^CD127^−^FoxP3^+^ of Treg final products.
4. Correlation between ALSFRS-R score and Treg inhibitory function: ALSFRS-R score and Treg cell products showed an overall negative correlation trend in inhibiting PBMC proliferation. The ALSFRS-R score showed a moderate positive correlation with the ability of Treg cell products to inhibit IFN-γ secretion at 1:1 and 1:2, with higher ALSFRS-R scores indicating better inhibition of IFN-γ secretion.

### 3.4 Phenotypic analysis of Treg products from cryopreserved healthy donors, fresh healthy donors, and ALS patients

**Figure 4:**
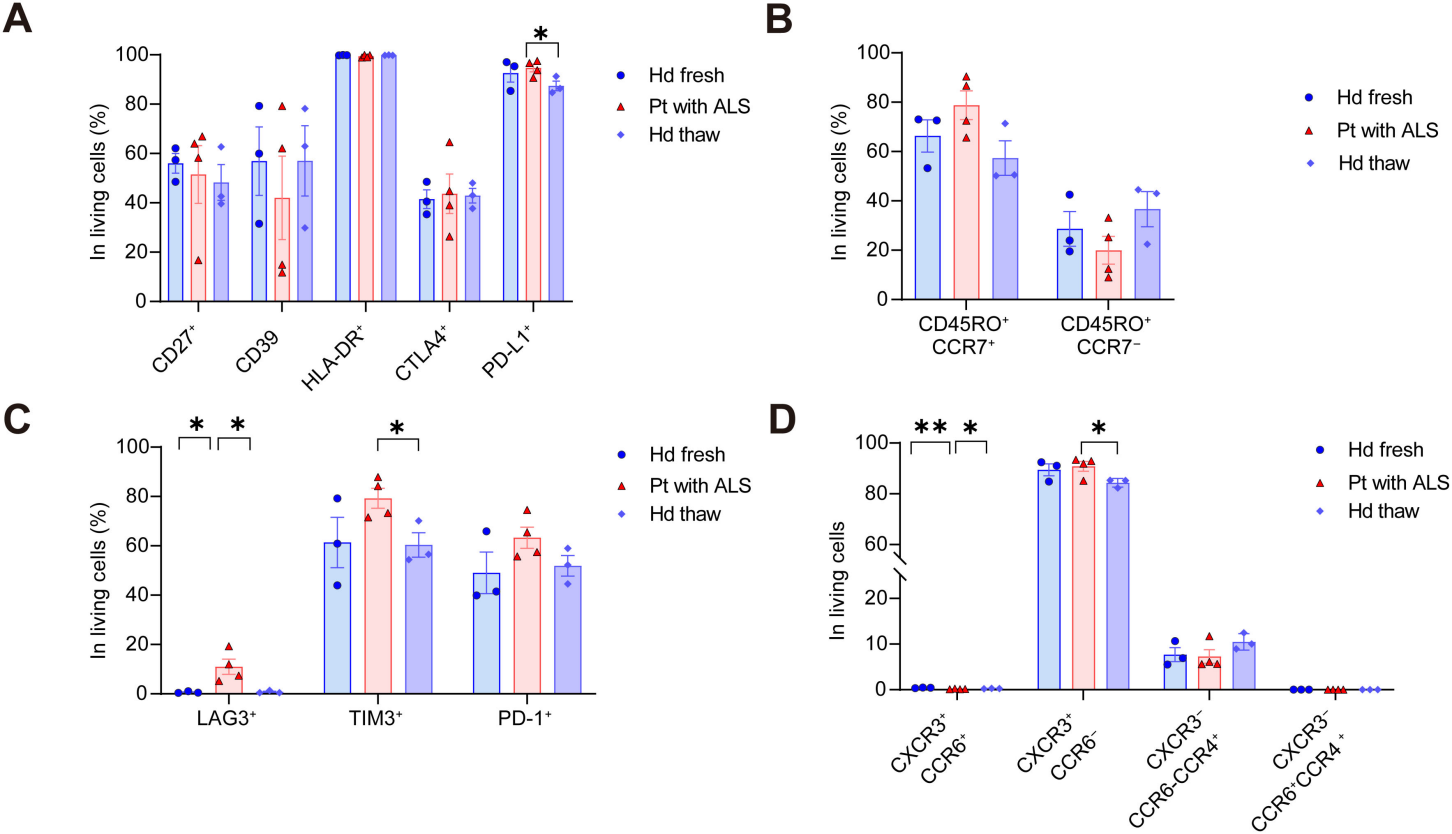
Comparative analysis of phenotypic profiles of GMP - grade Treg products between healthy donors (fresh or thaw) and patients with amyotrophic lateral sclerosis, HD fresh: n = 3, HD thaw: n = 3, ALS: n = 4, graphs show mean ± SEM. **(A)** Functional phenotypic characterization of treg cells **(B)** Cell sub setting markers. **(C)** LAG3\TIM3\PD1 markers **(D)** Th - like phenotypic markers. (*P<0.05; **P <0.01).

In this study, the effects of healthy donor fresh APH (n = 3, HD fresh), cryopreserved APH (n = 3, HD thaw) and patient fresh APH (n = 4, Pt fresh) on the expression of functionally relevant phenotype in Treg products were systematically evaluated. The results showed (**Figure 4A**): CD39 expression in ALS patients showed a trend of decrease compared to healthy donors, but there was no significant difference; The expression of PD-L1 of ALS patient was significantly higher than HD derived with fresh APH (*P* < 0.05). There was no significant difference in the expression ratios of other key functional phenotype CTLA4, CD27^+^ and HLA-DR^+^. Memory subset analysis showed (**Figure 4B**) that CD45RO^+^CCR7^+^ (central memory T cells, Tcm) of ALS group was higher, and CD45RO^+^CCR7^−^ (effector memory T cells, Tem) was lower, but there was no significant difference from the healthy APH group (*P* > 0.05).

The expression of LAG3^+^, TIM3^+^, PD-1^+^ was higher in ALS patients than in healthy donors, and there were significant differences of LAG3 and TIM3(**Figure 4C**). After sorting, the expression levels of LAG3 and TIM3 were detected in Tregs isolated from ALS patients and healthy donors, to confirm that the upregulation of these molecules emerged during *in vitro* expansion. The results revealed that the expression of LAG3 and TIM3 remained low in freshly sorted Tregs from both ALS patients and healthy donor, with no significant intergroup differences (**Supplementary Figure 3**).

Following *in vitro* expansion, TIM3 expression was significantly increased in Treg cells from both ALS patients and healthy donors, while LAG3 expression was also significantly upregulated in Treg cells derived from ALS patients. Notably, the function of Treg cells is distinctly different from that of chimeric antigen receptor T (CAR-T) cells, which have been extensively studied in recent years. PD-1, LAG3, and TIM3 are all classic immune checkpoint molecules, and in Treg cells, these molecules play a crucial role in assisting Treg cells to exert their immunosuppressive function. The upregulation of their expression after *in vitro* expansion is a desirable result, indicating that Treg cells were effectively activated.

Previous studies have shown that LAG3^+^TIM3^+^ Treg cells exhibit stronger immunosuppressive capacity than LAG3^−^TIM3^−^ Treg cells (31). The higher expression of TIM3 and LAG3 in Treg cells derived from ALS patients compared with those from healthy donors can be explained as follows: Treg cells in ALS patients undergo a more intense state of confrontation against immune imbalance *in vivo*; when reactivated *in vitro*, they tend to exhibit stronger immunosuppressive function than Treg cells from healthy donors.

Consistently, other studies have reported that TIM3 expression is higher in Treg cells within the tumor microenvironment, which is associated with enhanced immunosuppressive function of Treg cells (32). In addition, LAG3 is also highly expressed in a variety of autoimmune diseases, however, its functional significance in these diseases remains unclear (33).

The analysis of chemotaxis and homing receptors showed that that the proportion of Th1-like Treg cells (CXCR3^+^CCR6^−^) in the product was the highest (**Figure 4D**).

### 3.5 mRNA sequencing reveals different transcription profiles of expanded Treg cells

**Figure 5:**
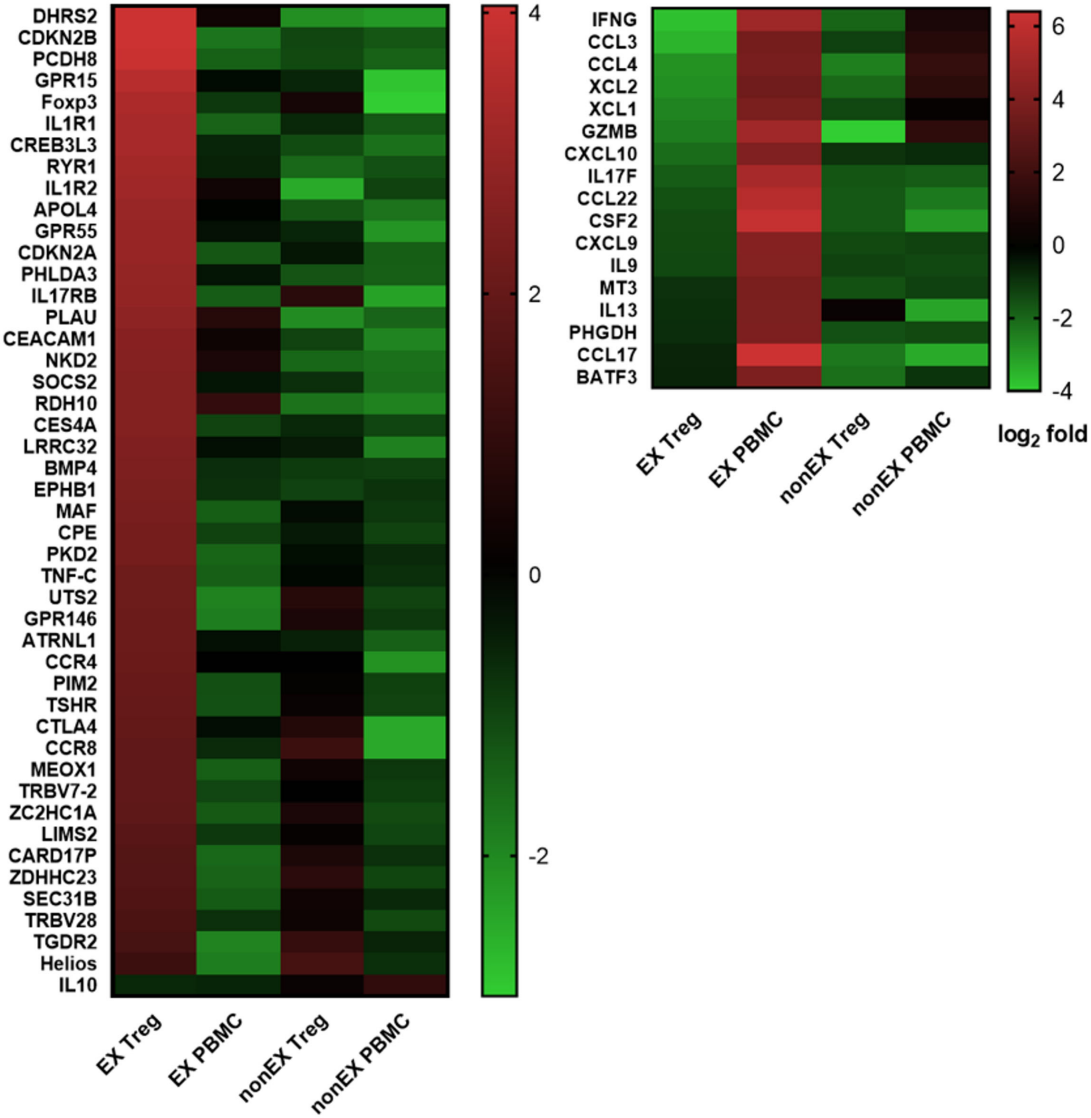
mRNA sequencing of four cell populations: expanded Treg cells, expanded PBMCs, non-expanded Treg cells, and non-expanded PBMCs (n = 3 biological replicates per group). **(A)** Transcription factor and pathway-related panel: The genes with high expression in expanded Tregs and log_2_ fold change (log_2_FC) values greater than 1 (IL10 was included due to research concerns). **(B)** Inflammatory factor-related panel: Pro-inflammatory and chemokine-related genes of clinical interest in subsequent studies.

To confirm the safety and identity of expanded Treg cells by distinguishing them from inflammatory cells, and to assess the transcriptional impact of ex vivo expansion, we performed batch mRNA sequencing of four cell populations: expanded Treg cells, expanded PBMCs, non-expanded Treg cells, and non-expanded PBMCs (n = 3 biological replicates per group). **Figure 5A** displays genes that are highly expressed in expanded Treg cells with a log₂ fold change greater than 1. **Figure 5B** shows pro-inflammatory and chemokine-related genes of clinical interest in subsequent studies.

As shown in **Figure 5B**, expanded Treg cells form a transcriptionally coherent cluster that is highly consistent with non-expanded Treg cells and distinctly different from expanded PBMCs in transcriptional profiles, indicating that the expansion procedure maintains the lineage fidelity of Treg cells. Expanded Treg cells consistently exhibited low expression of pro-inflammatory mediators such as IFNγ, IL-17, IL-9, IL13, GZMB and CSF2, which were significantly elevated in the expanded PBMCs. CCL3/CCL4 (MIP-1α/β): Pro-inflammatory chemokines that recruit monocytes/macrophages and T cells and are involved in inflammation and the tumor microenvironment. XCL1/XCL2: Chemokines that chemoattract T cells and dendritic cells (DCs), participating in immune cell recruitment and lymphoid organ development. CXCL9/CXCL10: IFN-γ-inducible chemokines that recruit Th1 cells, involved in antiviral and antitumor immunity. CCL17/CCL22: Chemokines that chemoattract Tregs and Th2 cells, participating in immune tolerance and allergic responses. These chemokines were highly expressed in expanded PBMCs, while their expression remained low in both non-expanded and expanded Treg cells.

**Figure 5A** displays genes that are highly expressed in expanded Treg cells with a log₂ fold change greater than 1. Except for Helios and IL10, these genes are highly expressed in expanded Tregs compared with the other three groups, and there is a significant difference between them and expanded PBMCs.

Among these, the core genes responsible for exerting immunosuppressive functions and maintaining self-stability in regulatory T cells (Treg cells) are as follows:

FOXP3, known as the “master” transcription factor of Treg cells, is indispensable for the development, differentiation, and functional exertion of Treg cells. It modulates the expression of numerous other Treg-associated genes, such as CTLA4 and CD25. Foxp3 was undetectable in PBMCs regardless of expansion status. It was weakly expressed in non-expanded Treg cells, and cellular expansion significantly upregulated Foxp3 expression in Treg cells. Combined with the stability data, the proportion of Foxp3^+^ cells did not show a downward trend in the 15-month test of two batches; instead, it fluctuated within a certain range, which was associated with different donors as well as the accuracy and stability of the flow cytometry detection method. Interleukin-2 (IL-2) was added during the *in vitro* expansion process. Specifically, IL-2 activates the STAT5 signaling pathway, which directly binds to the promoter and enhancer regions of the Foxp3 gene to promote Foxp3 transcription. Rapamycin can inhibit the transformation of Tregs into pro-inflammatory phenotypes by suppressing the mTOR signaling pathway, reducing the activation of pro-inflammatory transcription factors (e.g., STAT3), while directly enhancing the transcriptional activity and stability of Foxp3, thereby further promoting its high expression. Additionally, rapamycin synergizes with IL-2 to maintain the functional characteristics of expanded Tregs. Leucine-rich repeat-containing protein 32 (LRRC32, also known as GARP) is expressed on the surface of activated Tregs; it plays a crucial role in activating transforming growth factor-β (TGF-β) and is a key molecule mediating the inhibitory function of Tregs. TGF-β can induce the differentiation of naive T cells into Tregs and stabilize Foxp3 expression to prevent its degradation. Furthermore, transcription factors such as MAF, which are highly expressed in post-expansion Tregs, can synergize with Foxp3 to promote its own expression and maintain the cellular properties of Tregs.

CTLA4 (Cytotoxic T-Lymphocyte-Associated Protein 4) serves as a key inhibitory receptor. It suppresses the initiation of immune responses by binding to CD80/CD86 molecules on the surface of antigen-presenting cells (APCs) and transmitting inhibitory signals into the cells. IL1R2 (Interleukin-1 Receptor Type 2) acts as an antagonist of the interleukin-1 (IL-1) receptor. As a “decoy” receptor, it binds to IL-1 specifically, thereby sequestering IL-1 and inhibiting its pro-inflammatory signaling pathways.

Receptors Related to Treg Cell Migration: CCR4 & CCR8: These are chemokine receptors responsible for guiding Treg cells to migrate to specific tissues or inflammatory sites, enabling them to exert local immunosuppressive effects. GPR15 & GPR55: They belong to G protein-coupled receptors. GPR15 is considered to mediate the migration of Treg cells to barrier tissues such as the gut and skin. Both receptors are involved in cell-to-cell adhesion and may participate in the migration and tissue localization of Treg cells. These two chemokines suggest that intravenously infused Treg cells tend to migrate toward the intestines and skin, indicating that such Treg-based therapy may achieve better therapeutic efficacy in corresponding autoimmune diseases.

Genes Related to Treg Cell Proliferation and Activation:CDKN2A & CDKN2B: These are cell cycle inhibitory proteins, encoding p16 and p15, respectively. They inhibit cell cycle progression and regulate cell proliferation, and their expression may be associated with the resting or senescent state of Treg cells. PD-1 signaling can directly upregulate CDKN2B, which is consistent with the flow cytometry data, suggesting that excessive expansion duration may contribute to this phenomenon. PIM2: A serine/threonine kinase that promotes cell survival and proliferation in Treg cells. BMP4 (Bone Morphogenetic Protein 4): Belonging to the TGF-β superfamily, it may be involved in regulating the differentiation or functional maintenance of Treg cells. PHLDA3: It regulates the Akt signaling pathway, thereby affecting the survival and metabolism of Treg cells. RDH10 (Retinol Dehydrogenase 10): It is involved in the synthesis of retinoic acid, a metabolite of vitamin A, which is an important factor inducing the differentiation of peripheral Treg cells (pTregs). CEACAM1 (Carcinoembryonic Antigen-Related Cell Adhesion Molecule 1): As a cell adhesion molecule, it transmits inhibitory signals and participates in the regulation of T cell activation and function, including Treg cells.

The expression level of Helios in Treg cells after amplification was lower than that before amplification, indicating that peripheral Treg cells (pTregs) are more prone to proliferation during the amplification process.

Other genes are less studied or have weaker associations with T cells, making it impossible to determine their correlation with Treg cells.

These results suggest that the amplification protocol not only preserves but further enhances the inhibitory transcriptional phenotype of Treg cells, thereby minimizing the risk of inflammatory skew.

Taken together, these transcriptional data indicate that *ex vivo* expansion of Treg cells maintains and enhances lineage-defining gene expression, maintains high levels of regulatory markers (FoxP3, Helios, CTLA4), and effectively inhibits the production of pro-inflammatory cytokines. These properties support the stability and safety of expanded Treg cells, providing a solid foundation for their application in immunotherapy settings.

### 3.6 Animal pharmacodynamics

The therapeutic efficacy of Treg cells was investigated in an ALS spontaneous mouse model. Compared with control mice, those treated with mouse Tregs exhibited a significant delay in ALS disease onset and a trend towards prolonged overall survival. The biological efficacy of Treg cells was also studied using a GvHD mouse model reconstituted with human PBMCs. Compared with control mice, those in the Treg-treated group showed significantly prolonged survival, improved weight loss, and a trend towards reduced GvHD severity.

Pharmacodynamic validation was carried out in both GvHD and ALS models, establishing a stable technical basis for personalized Treg immunotherapy for ALS. This process has now progressed to clinical trials. (NCT06671236).

### 3.7 Validation in SOD-1 mutant ALS animal model

**Figure 6:**
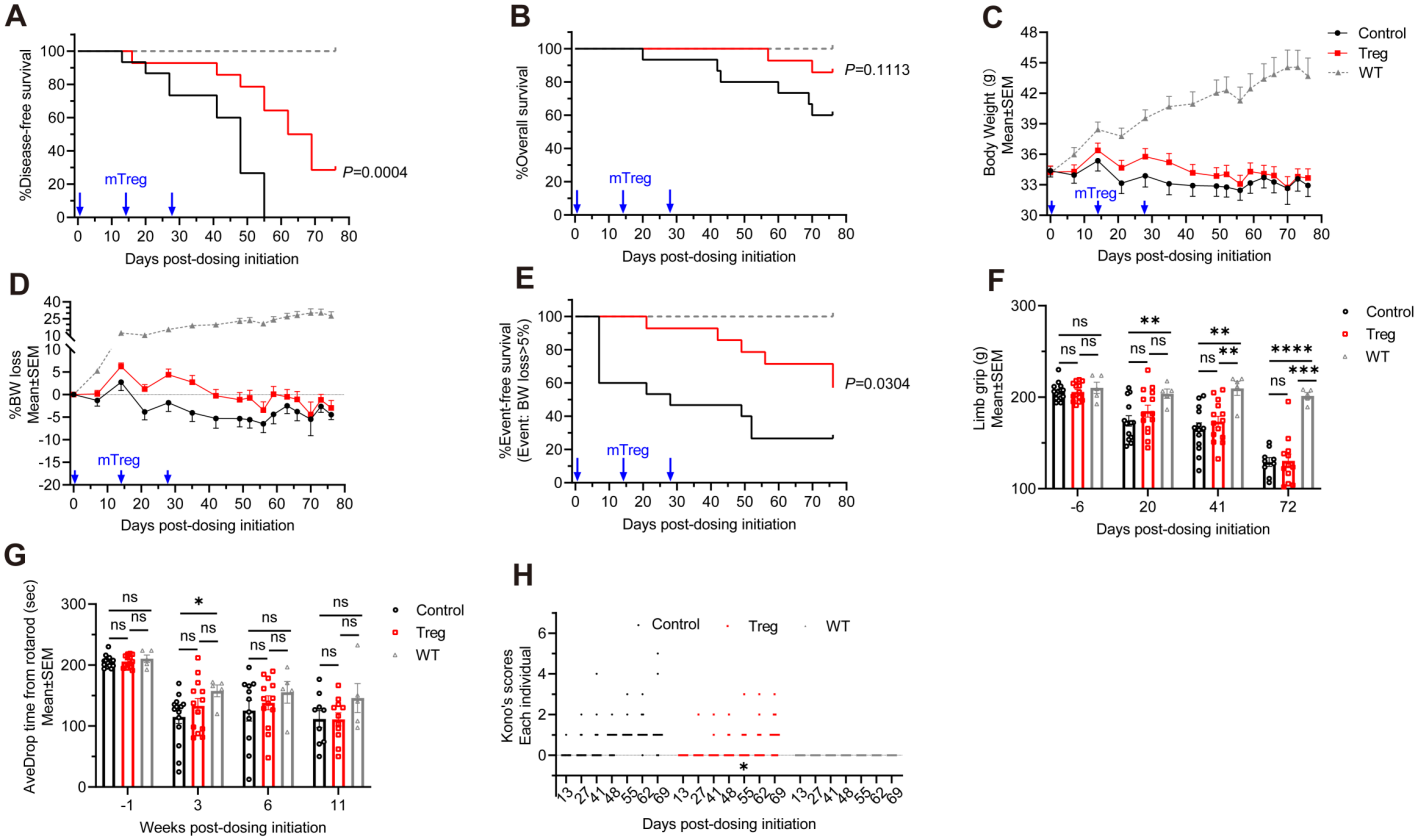
*In vivo* efficacy of murine Treg on SOD1 mice. **(A)** Disease-free survival. **(B)** Overall survival. **(C)** Body weight. **(D)** Body weight loss. **(E)** Event-free survival. **(F)** Limb grip strength **(G)** Drop time from rotarod. **(H)** Kono’s score. (*P <0.05; **P <0.01; ***P <0.001; ****P <0.0001). ns, No signification.

The biological efficacy of Tregs was evaluated in a spontaneous mouse model of ALS, using Tregs isolated from donor mouse splenocytes as surrogate cells. Compared with control mice, mice treated with mouse Tregs showed a significant delay in ALS disease onset (*P* = 0.0004), (**Figure 6A**), a trend of prolonged overall survival, not statistically significant (*P* = 0.1113), (**Figure 6B**), and a trend of delayed weight loss during the administration phase (D0-D28) (**Figure 6C**). Event-free survival (EFS), which is defined as the day an individual first loses > 5% of their body weight compared to baseline. (**Figure 6D**), the EFS of mice receiving Treg administration was significantly prolonged compared to the control group (*P* = 0.0304) (**Figure 6E**). No statistical significance was observed in the Treg administration group and the control group for motor function (dwell time on the rotating rod and hindlimb grip) in mice, showing a certain trend of delayed residence time and enhanced hindlimb grip (**Figures 6F, G**). The Kono score of the control and Treg mice increased over time, as shown in **Figure 6H**. However, compared with the control group, the mice receiving Treg administration showed a tendency to increase the delay in ALS Kono scores, which was reflected in the fact that fewer animals scored > 0 and/or had lower mean scores within the group, significant on D55 (*P* = 0.011), and this indicator decreased over time as the disease progressed in both groups. (**Figure 6H**).

### 3.8 Validation in animal GvHD model

**Figure 7:**
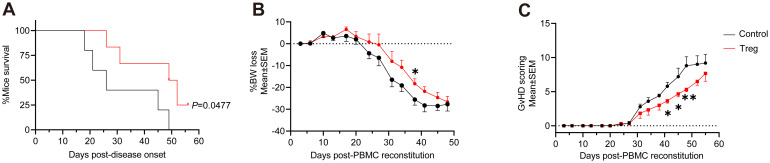
*In vivo* efficacy of human Treg on GvHD mice. **(A)** Survival from disease onset. **(B)** Body weight loss. **(C)** GVHD scores. (*P <0.05; **P <0.01).

The biological efficacy of Treg cells prepared according to the protocol described in this article was investigated using a GvHD mouse model induced by human PBMC reconstruction. The mice in the Treg cell therapy group showed a significant increase in survival compared to the control group. (*P* < 0.05), (**Figure 7A**), and the improvement trend in weight loss was significant on D38. (*P* < 0.05, **Figure 7B**). The severity of GvHD improved with a significant difference between the treatment group and the control group from D41 to D48. (*P* < 0.05 or *P* < 0.01), (**Figure 7C**). Tregs can only inhibit the subsequent expansion of effector T cells, but cannot reverse established tissue injury and inflammatory pathology, thus only delaying disease progression was observed. Since mouse models cannot fully recapitulate the human physiological microenvironment, the detectable therapeutic trend at the early efficacy exploration stage is sufficient to support further in-depth research.

## 4 Discuss

Currently, there are limited treatment options for ALS, including Riluzole, Edaravone, and Tofersen for patients with SOD-1 mutant ALS. Regardless of the underlying cause, ALS progression is associated with immune dysregulation, including abnormal astrocyte activation, microglial polarization toward the pro-inflammatory M1 phenotype, and infiltration of peripheral immune cells into the CNS. Treg cells can recruit oligodendrocytes to promote myelin regeneration while regulating the immune response and play a role in delaying disease progression and certain disease recovery.

Several companies worldwide have explored Treg-based therapies for ALS, including autologous peripheral venous refusion, umbilical cord blood-derived allogeneic intravenous refusion, and Treg production methods and pediatric surgical waste thymus sources. In terms of safety and accessibility, peripheral blood–derived sources are the most convenient, and autologous infusion of immune cells remains the safest approach. However, we are currently developing autologous Treg cells. Given the low frequency of endogenous Tregs *in vivo*, approximately 20 days are required for ex vivo expansion, together with more than ten days for quality release procedures. Therefore, this therapy is not applicable for acute diseases. In addition, the overall treatment cost remains relatively high. Some patients with severe ALS cannot take enough peripheral blood to meet the initial requirements of the process. Therefore, to solve this part of the clinical need, it is necessary to develop allogeneic Treg cells. At present, there are cord blood-derived Treg cells that do not need to be matched for intravenous refusion treatment in ALS patients, but they have not been seen to be systematically studied for safety. The low immunogenicity of thymus-derived Treg cells has also not been verified. Therefore, Treg cells prepared from autologous peripheral sources will continue to be the mainstream treatment method during this period.

This study started with the sorting process of Treg cells, established a GMP-grade *in vitro* Treg culture system, verified the feasibility of cryopreservation APH as a starting material, and evaluated the therapeutic potential of Treg cells from ALS patients from *in vitro* functional verification, gene expression, and animal experiments.

The proportion of CD4^+^CD25^+^CD127^−^ cells of interest was higher than 95% on average, and the inhibitory function was strong. APH products can be used as starting material after cryopreservation and recovery, providing greater flexibility in Treg production. The expansion fold of Treg cells in ALS patients was lower than that of healthy donors, but there were no significant differences in phenotype and function. The results of the correlation study on ALS patient score and Treg suggested that the higher the ALSFRS-R (milder the disease), the higher the proportion of early Treg cells cultured in APH and Tregs after classification. After culture, this correlation disappears.

Treg cells produced by this system have also been used for the reinfusion treatment of ALS patients. It is worth noting that, given the inherent heterogeneity of Treg cell states among individual patients, there exists a theoretical risk of Treg expansion failure in clinical practice. However, in the current clinical experience involving approximately 20 ALS patients who have received Treg reinfusion, no cases of expansion failure have been observed to date. Nevertheless, this preliminary data is based on a small sample size, and further expansion of the sample size is essential to collect more robust data, verify the consistency of Treg expansion efficiency across different patient populations, and fully assess the potential risks of expansion failure in a larger cohort. The current clinical use method is to inject every 28 days, three injections. The ALS score remained stable during administration and did not decrease, with a decrease in score three months after the last injection.

## Data Availability

All data supporting the findings of this study are included in the article and its supplementary materials. Further inquiries can be directed to the corresponding author.

## Supplementary material

Supplementary Figure 1 Differences cell viability of Treg cell products by different cell sorting methods (FACS-Protocol A, MicroBeads-Protocol B, Kit-Protocol C) after sorting. n = 3, Graphs show Mean ± SEM. Sorting phase: Cell viability.

Supplementary Figure 2 Treg cells were thawed and assessed for recovery at 1, 2, 3, 6, 9, 12, and 15 months after cryopreservation. n=2. **(A)** Osmolality; **(B)** pH; **(C)** Cell viability; **(D)** Treg purity; **(E)** Suppression; **(F)** Cell density; **(G)** Foxp3^+^; **(H)** Non-Treg cell.

Supplementary Figure 3 Comparative analysis of phenotypic profiles of LAG3 and TIM3 after sorting between Healthy donors and Patients with Amyotrophic Lateral Sclerosis, HD: n = 3, ALS: n = 3, Graphs show Mean ± SEM. **(A)** LAG3 and TIM3 of Treg cells after sorting but before *in vitro* culture.
