# Supplementary figures and images for: Development and efficacy of ex vivo expanded autologous regulatory T cells for the treatment of amyotrophic lateral sclerosis
Source: Front Immunol. 2026 Jul 2;17:1854252. doi: 10.3389/fimmu.2026.1854252 (PMC13372620; doi:10.3389/fimmu.2026.1854252)

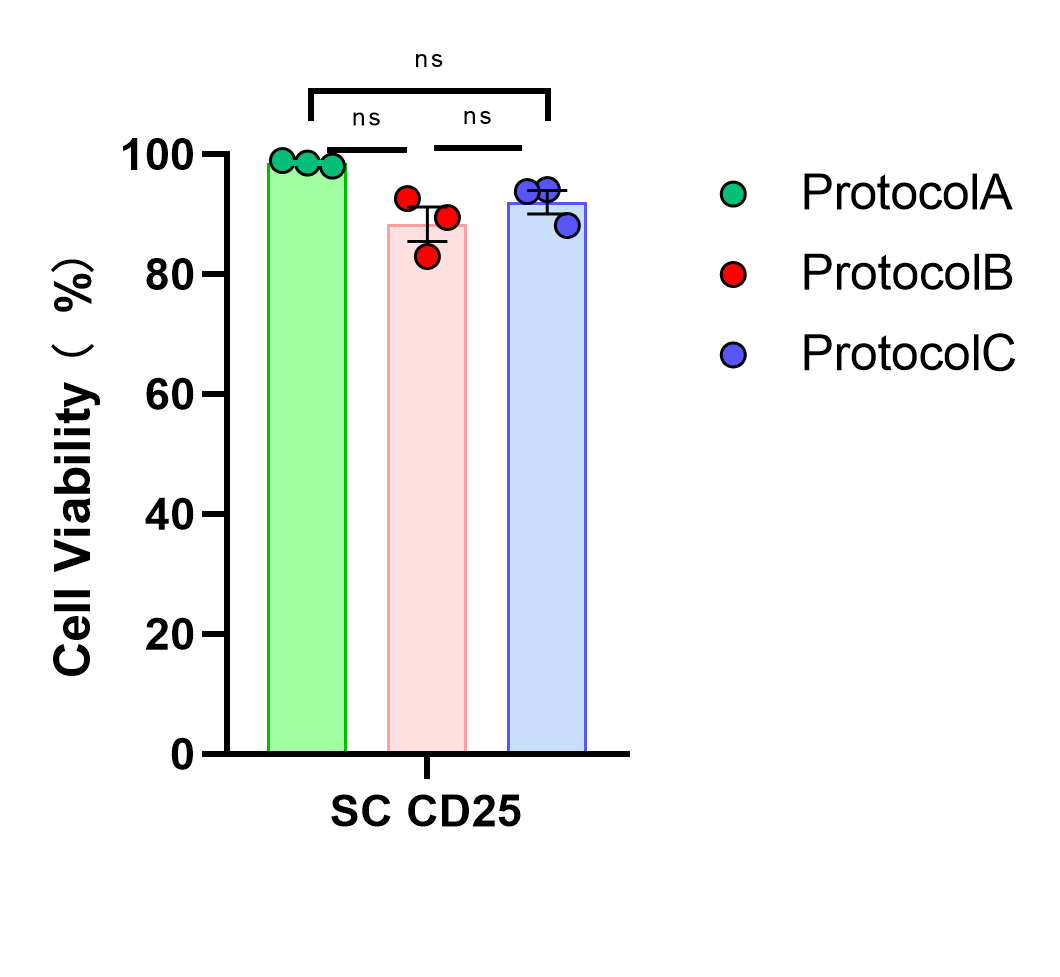

Supplement: Supplementary Figure 1 — Differences cell viability of Treg cell products by different cell sorting methods (FACS-Protocol A, MicroBeads-Protocol B, Kit-Protocol C) after sorting. n = 3, Graphs show Mean ± SEM. Sorting phase: Cell viability. [file Image1.tif]

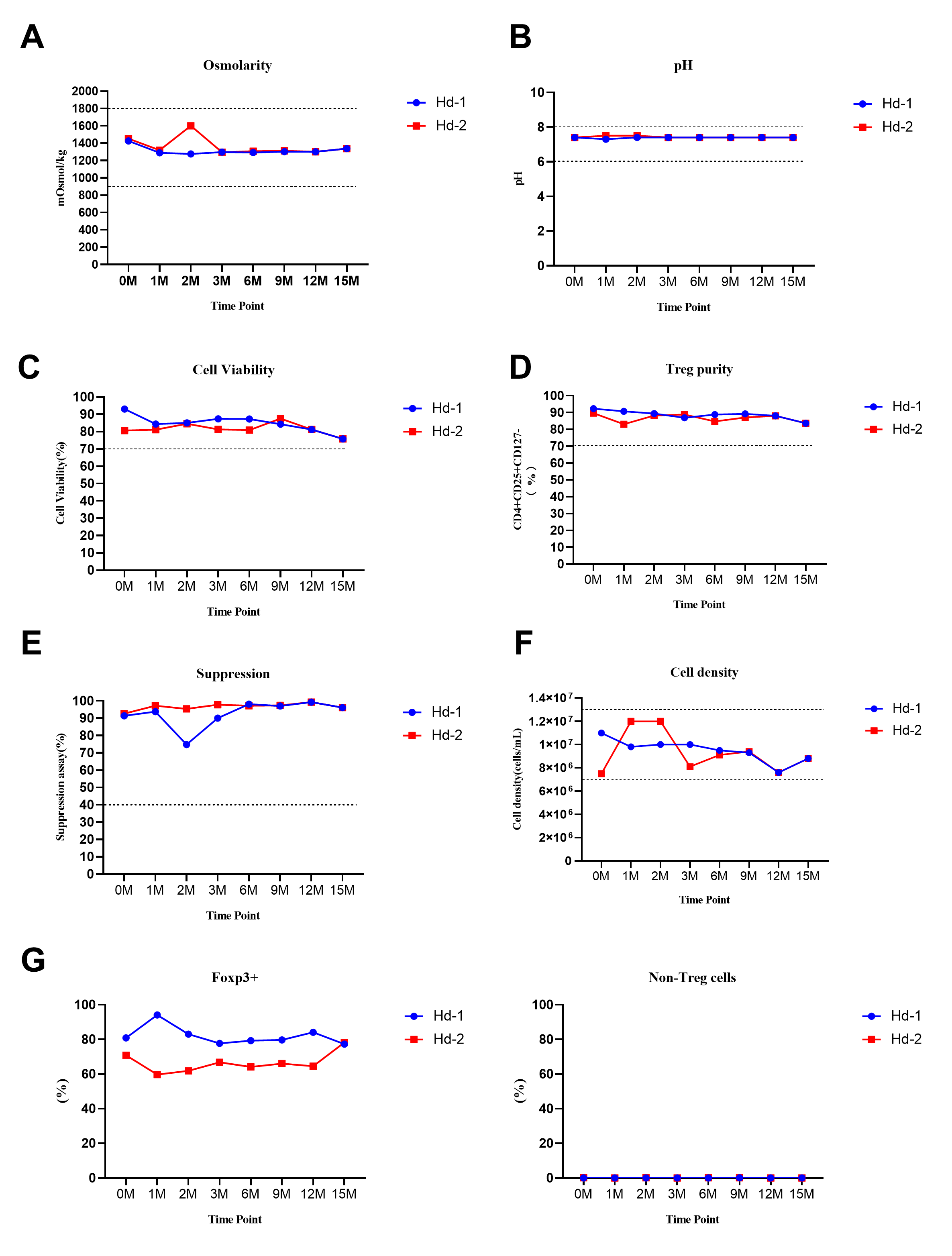

Supplement: Supplementary Figure 2 — Treg cells were thawed and assessed for recovery at 1, 2, 3, 6, 9, 12, and 15 months after cryopreservation. n=2. (A) Osmolality; (B) pH; (C) Cell viability; (D) Treg purity; (E) Suppression; (F) Cell density; (G) Foxp3+; (H) Non-Treg cell. [file Image2.tif]

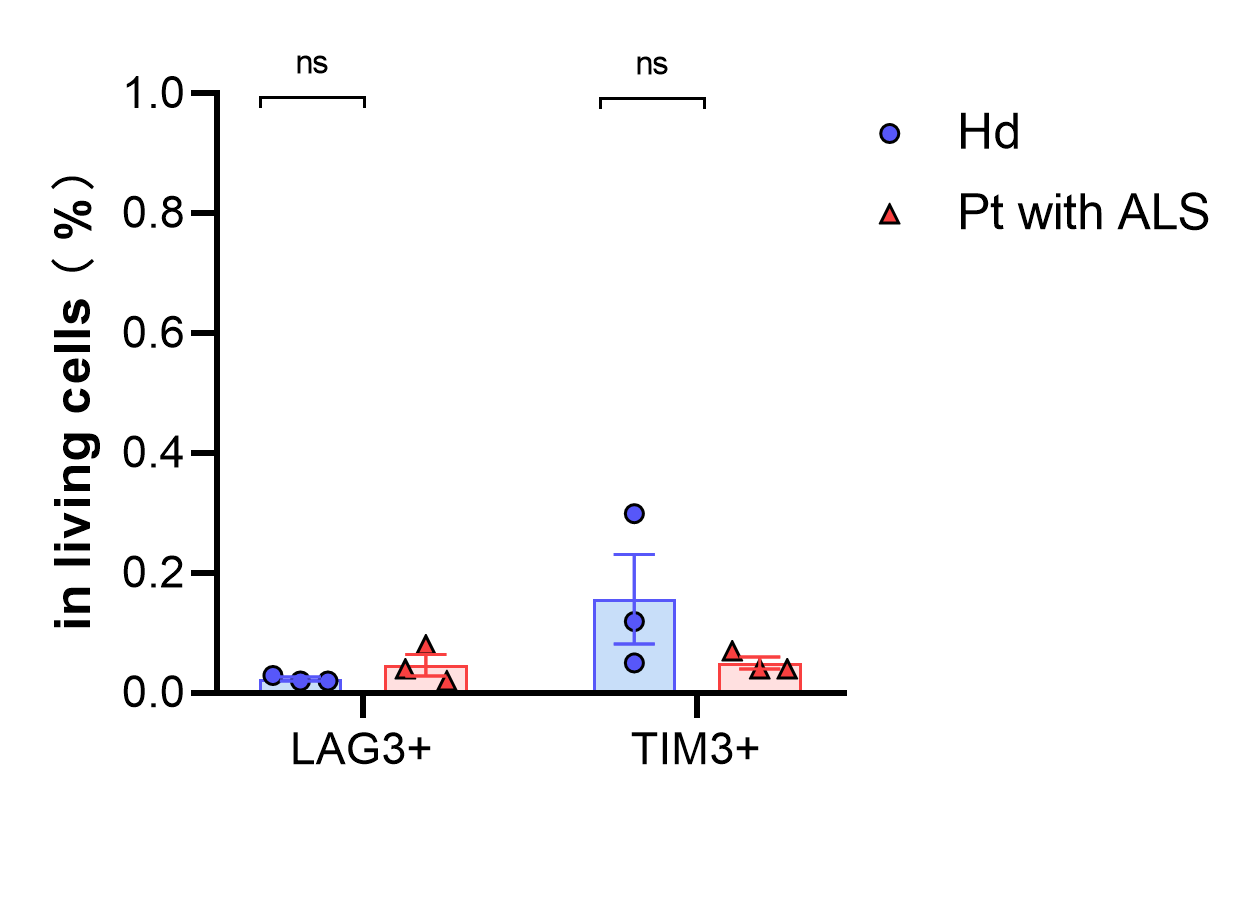

Supplement: Supplementary Figure 3 — Comparative analysis of phenotypic profiles of LAG3 and TIM3 after sorting between Healthy donors and Patients with Amyotrophic Lateral Sclerosis, HD: n = 3, ALS: n = 3, Graphs show Mean ± SEM. (A) LAG3 and TIM3 of Treg cells after sorting but before in vitro culture. [file Image3.tif]
